# Chronic Neurobehavioral Sex Differences in a Murine Model of Repetitive Concussive Brain Injury

**DOI:** 10.3389/fneur.2019.00509

**Published:** 2019-05-22

**Authors:** Laura B. Tucker, Alexander G. Velosky, Amanda H. Fu, Joseph T. McCabe

**Affiliations:** ^1^Pre-Clinical Studies Core, Center for Neuroscience and Regenerative Medicine, Uniformed Services University of the Health Sciences, Bethesda, MD, United States; ^2^Department of Anatomy, Physiology & Genetics, F.E. Hébert School of Medicine, Uniformed Services University of the Health Sciences, Bethesda, MD, United States

**Keywords:** traumatic brain injury, mouse, concussion, repetitive brain injury, behavior, active place avoidance, microbleeds, sex differences

## Abstract

Traumatic brain injury (TBI) resulting from repeated head trauma is frequently characterized by diffuse axonal injury and long-term motor, cognitive and neuropsychiatric symptoms. Given the delay, often decades, between repeated head traumas and the presentation of symptoms in TBI patients, animal models of repeated injuries should be studied longitudinally to properly assess the longer-term effects of multiple concussive injuries on functional outcomes. In this study, male and cycling female C57BL/6J mice underwent repeated (three) concussive brain injuries (rCBI) delivered via a Leica ImpactOne cortical impact device and were assessed chronically on motor (open field and rotarod), cognitive (y-maze and active place avoidance), and neuropsychiatric (marble-burying, elevated zero maze and tail suspension) tests. Motor deficits were significant on the rotarod on the day following the injuries, and slight impairment remained for up to 6 months. All mice that sustained rCBI had significant cognitive deficits on the active place avoidance test and showed greater agitation (less immobility) in the tail suspension test. Only injured male mice were significantly hyperactive in the open field, and had increased time spent in the open quadrants of the elevated zero maze. One year after the injuries, mice of both sexes exhibited persistent pathological changes by the presence of Prussian blue staining (indication of prior microbleeds), primarily in the cortex at the site of the injury, and increased GFAP staining in the perilesional cortex and axonal tracts (corpus callosum and optic tracts). These data demonstrate that a pathological phenotype with motor, cognitive, and neuropsychiatric symptoms can be observed in an animal model of rCBI for at least one year post-injury, providing a pre-clinical setting for the study of the link between multiple brain injuries and neurodegenerative disorders. Furthermore, this is the first study to include both sexes in a pre-clinical long-term rCBI model, and female mice are less impaired functionally than males.

## Introduction

The effects of traumatic brain injury (TBI) are far-reaching, with a global incidence estimated in 2007 to be ~10 million (1). In 2013, there were ~2.5 million TBI-related emergency room visits in the United States alone (2). Although the majority of sustained TBIs are mild and classified as concussions (concussive brain injury; CBI), many of the millions of the survivors suffer persistent post-injury complications that include cognitive, motor, and neuropsychiatric symptoms, and in some cases these issues can persist for over 10 years (3).

In recent years, more attention has been directed to the effects of repeated TBI, particularly in the context of military operations and contact sports (e.g., American football, soccer, boxing). Both military personnel and contact sports participants are at greater risk for exposure to multiple concussive and sub-concussive TBIs (repeated concussive brain injuries; rCBI). A recent study estimated the incidence of TBI in Iraq and Afghanistan veterans to be ~17%, with half sustaining multiple head injuries (4). In the context of sports, head injuries are associated with multiple contact sports in both men and women (5), and in the National Football League up to 30% of players sustaining a concussion go on to receive repeat TBIs (6). Multiple TBIs are associated with delayed neurodegenerative conditions including chronic traumatic encephalopathy (7), a condition characterized by perivascular accumulation of hyperphosphorylated tau (8) and behavioral symptoms including (but not limited to) cognitive dysfunction, violence, depression, and suicidality (9, 10).

As there are no fully effective therapies to treat TBI, or rCBI specifically, translational studies remain of importance. Animal models that replicate at least a subset of the pathological and behavioral symptoms that are observed following clinical rCBI create a context in which therapies can be tested. Many functional symptoms (i.e., cognitive and neuropsychiatric problems) may not appear in human TBI patients for months or years following sustained injuries, and there is a critical need to develop chronic animal models with persistent functional and neuropathological symptoms to provide a context in which delayed treatments can be assessed. Many rodent models of rCBI have been developed and described, and many have clinical relevance with behavioral symptoms similar to rCBI patients, including cognitive deficits and/or neuropsychiatric changes and axonal damage [e.g., (11–16)]. However, most of the observations have been limited to more acute time periods (days, or up to a few months) following the injuries.

There are a small number of rCBI studies in mice that have extended observations to a year or longer following injuries and continue to report functional and neuropathological effects of rCBI (17–20). These studies, however, have been limited to male mice. Clinically, female athletes are at equal or greater risk of sustaining head injuries than males during participation in sports (21–26), and although many studies have demonstrated a poorer clinical outcome in women following CBI [e.g., (27, 28)], data remain inconsistent (29). Thus, there is a continued need to be inclusive of both sexes in pre-clinical rCBI research.

The goal of this study was to study sex differences in behavioral symptoms over a chronic period in a murine model of rCBI that results in axonal damage. Although prior studies included both male and female rodents in TBI behavioral studies [e.g., (15, 30–33)], most have employed the controlled cortical impact (CCI) brain injury model and evaluated outcomes at more acute time points. The injury model employed in this study has an advantage of being less invasive than traditional CCI and fluid percussion injury models that require craniectomy. Although this injury method does not include rotational acceleration, an important component in a majority of clinical head trauma cases, CBI allows for relatively consistent control of the mechanical effects of impact injury, which can be a disadvantage in at least some rotational injuries where there is no precise control of rotational effects due to inherent differences in head response.

## Methods

### Animals and Housing

All animal procedures were approved by the Institutional Animal Care and Use Committee at the Uniformed Services University of the Health Sciences (Bethesda, MD). Male and female mice, 8 weeks old, were obtained from Jackson Laboratories (C57BL/6J, 000664; Bar Harbor, Maine) and group-housed in Association for Assessment and Accreditation of Laboratory Animal Care-Accredited facilities with a standard 12-h light-dark cycle, with food and water available *ad libitum*. Animals acclimated to facilities for at least 1 week prior to baseline behavioral testing.

### Repetitive Concussive Brain Injury (rCBI) Procedures

Mice were randomly assigned to sham or injury groups. Mice in the injured group received three injuries at 24-h intervals. Concussive brain injury methods were performed as described by Velosky et al. (15). Briefly, mice were anesthetized with isoflurane and moved to a stereotaxic frame with anesthesia maintained via a flow-through nose cone. All fur was removed from the scalp with depilatory cream, Bregma was visualized under the skin with bright illumination and marked by a small dot with permanent marker. Anesthesia was discontinued with continuation of 100% oxygen, and a Leica ImpactOne impactor was immediately employed to deliver the injury to the scalp over parietal cortex (2.5 mm posterior to bregma and 2.5 mm left of bregma), with a 5-mm impact tip, 1.5 mm depth, 5.0 m/s velocity. Sham-treated mice underwent all procedures including the same duration of anesthesia, but the impactor was not activated.

### Apnea and Righting Reflexes

Immediately following concussive impact (rCBI mice) or discontinuation of anesthesia (sham controls) each day, any occurrence of cessation of breathing was measured and recorded as duration of apnea. Following injury or sham procedures, each mouse was placed into an individual clean cage over a warming pad in a supine position, and the time to return to a prone position was recorded as the righting reflex. Each day after all injury and sham procedures were complete mice were returned to group-housing.

### Body Weight Measurements

Baseline body weights were taken on the first day of injury, and mice were weighed at each behavioral testing time and on the day of euthanasia. Post-injury body weights are expressed as a percent of baseline measurements.

### Behavioral Testing

The number of mice in each sex and injury group at each behavioral testing time point are listed in Table 1. Two male and two female mice died due to rCBI procedures. Some mice were lost throughout the year of the study prior to the final behavioral test (tail suspension test), but the deaths were spontaneous (unexplained), or veterinarian-recommended euthanasias due to skin lesions. Thus, injury could not be determined to increase mortality rate during the 1-year study period. Open field (OF) and rotarod testing were performed multiple times during the experimental period of 1 year (see below); elevated zero maze (EZM), marble burying test (MBT), y-maze, active place avoidance (APA), and tail suspension test (TST) were only performed once at the end of the experiment.

**Table 1:** Number of animals (*n*) in each group and mean body weights (expressed as a percent of baseline weight, measured on the first injury day) at each behavioral testing time point.

	**Post-injury Day 1**		**Post-injury Day 30**		**Post-injury Day 90**		**Post-injury Day 180**		**Post-injury Day 360**	
	***n***	**Weight (%)**	***n***	**Weight (%)**	***n***	**Weight (%)**	***n***	**Weight (%)**	***n***	**Weight (%)**
Male—Sham	19	98.51	19	113.49	19	132.76	19	152.92	19	187.66
Male—rCBI	19	91.05^**^	19	106.33^**^	19	124.81^**^	19	139.69^**^	18	169.60^**^
Female—Sham	17	101.36	17	117.17	17	125.16	17	144.50	17	182.14
Female—rCBI	21	93.74	21	111.25	20	130.52	20	152.63	17	186.67

** *Significant difference in weights between injured male and sham-treated male mice across all post-injury days (p = 0.0064)*.

The procedures for OF and rotarod testing are found in more detail in a previous publication (32). For 3 days prior to injury, mice were trained to perform on an accelerating rotarod (4–60 rotations/min over 3 min, three trials/day); average latency to fall from the rotating rod on the final day was recorded as baseline performance. Prior to the third day of rotarod baseline testing, mice were placed in a 40 × 40 cm OF box (~5 Lux) with opaque walls connected to Any-Maze software (Stoelting Co., Wood Dale, IL) that tracked the position of the animal for 20 min. The software reported the total distance traveled in the arena, as well as the distance traveled in the center zone (20 × 20 cm), expressed as a percentage of the total distance traveled. Baseline OF testing was performed prior to rCBI; mice were re-tested in the OF and on the rotarod (in that order) on Days 1, 30, 90, 180, and ~1 year following the final CBI.

The EZM and MBT were performed as previously described 1 year after the injury on the first and second days following rotarod and OF testing, respectively (33). The EZM (Stoelting) is an annular platform raised 50 cm above the floor, divided into two opposing quadrants that are darkened and enclosed (~200 Lux) and the remaining two quadrants exposed and with greater amounts of light (~1,600 Lux). Movements of the mice were tracked with Any-Maze software for 5 min. For the MBT, mice were placed in a clear Plexiglas box (45 cm long × 24 cm wide × 22 cm high) filled with wood shavings to a depth of 5 cm. Twelve glass marbles were placed in a rectangular shape 6.5–9.5 cm apart; mice were placed in the boxes for 30 min. At the end of the test session, an experimenter blinded to the injury status of the mice counted the number of marbles buried to at least 2/3 depth.

On the fifth day following rotarod and OF testing, the y-maze novel arm test of spatial episodic memory, based on rodents' preference for novelty (34), was performed. The y-maze (Stoelting) has three arms at a 120° angle to each other, with a triangular central zone. For the first 5-min trial, one of the arms is blocked and the mice can explore the remaining two arms. After a 2-h inter-trial interval, the mice are placed back into the apparatus and all three arms are available. A camera above the maze recorded the movements of the mouse and Any-Maze software calculated the percent of time the mouse spent in the novel (previously blocked) arm. A mouse will normally spend a greater percentage of time in the novel arm, given it remembers the apparatus from the first trial in which that arm was blocked.

Three days following the y-maze test, spatial learning was tested in the active place avoidance test (APA; BioSignal Group, Brooklyn, NY) using methods similar to those described by Sangobowale et al. (35). The mice were first habituated to the circular arena (40 cm diameter) for 10 min, after which they underwent four testing trials with approximately a 40-min inter-trial interval. During each 10-min trial, the arena rotated slowly (~1 rotation/min); a fixed 60° segment of the apparatus was software-defined such that if the mouse entered that zone for longer than 500 ms an electric shock was applied (0.2 mA, 500 ms, every 1.5 s until the mouse left the zone). Thus, the mouse had to keep moving with the arena to avoid being shocked. Visual cues placed on the room walls around the arena facilitated spatial learning. On the following day, the 60° shock zone was rotated 180° from its location the previous day, and the mice had to learn to avoid the new location in four, 10-min trials.

The final test performed approximately a year following the injuries, 4 days following APA, was the TST. This test employed the procedures described by Can et al. (36). Mice were suspended by their tails from laboratory benches with tape (12 mm wide, 24 cm long) attached approximately 1 cm from the tip of the tail. To prevent tail-climbing, a 4-cm length of hollow polycarbonate tubing (1.3 cm inner diameter; McMaster-Carr, Santa Fe Springs, CA; #8585K41) was placed around the base of the tail prior to the test. Mice remained suspended for 6 min; sessions were videotaped, and the amount of time spent immobile was later scored by an investigator blinded to the injury condition of the animals.

### Histology and Immunohistochemistry

Following all behavioral testing, mice were transcardially perfused with 4% paraformaldehyde and tissue was prepared for immunohistochemistry as previously described (15). Brains from 14 injured male and 15 injured female mice (and five sham mice from each sex) were processed for Prussian blue staining combined with pararosaniline nuclear stain to detect regions of previous brain hemorrhages (manufacturer's instructions were followed for staining of mounted sections; Sigma-Aldrich, St. Louis, MO; HT20). Six mice from each injury/sex group were randomly selected for glial fibrillary acidic protein (GFAP) staining of free-floating sections as previously described (15), using a mouse monoclonal antibody (1:500, Thermo Fisher Scientific, MS-280-P) and a biotinylated secondary antibody (1:500; AffiniPure Goat Anti-Mouse IgG [H + L], Jackson ImmunoResearch Laboratories, 115-065-003) with DAB staining solution (Vector Labs, SK-4100). Sections were mounted onto slides, allowed to air-dry overnight and coverslipped.

**Figure 1:**
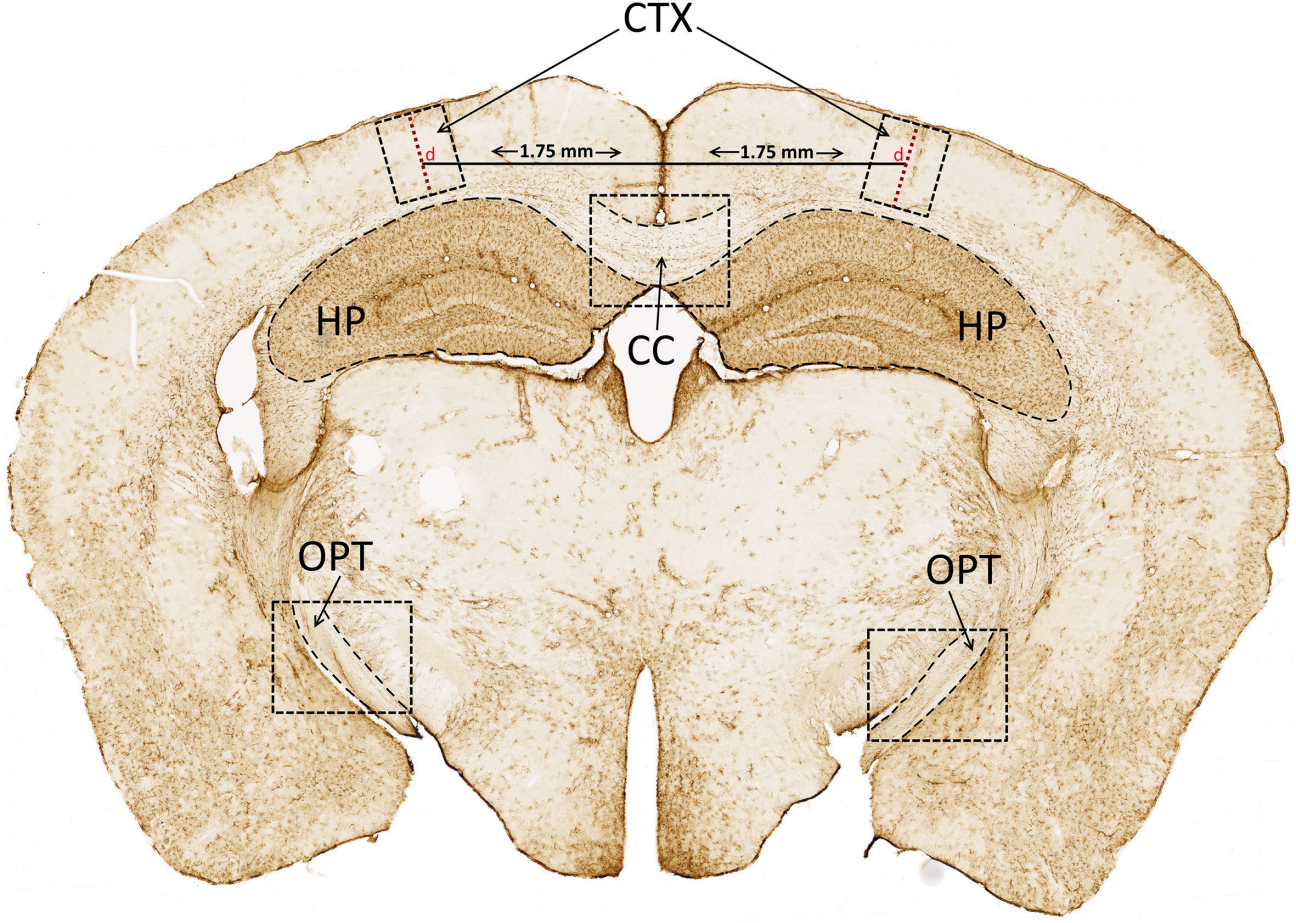
Cortical depth (d) and GFAP analysis. Cortical depth (d; red dashed line extending from surface of cortex to dorsal boundary of corpus callosum) was measured 1.75 mm from the superior sagittal fissure. GFAP staining density was analyzed in the CC, and bilaterally in the CTX, HP, and OPT. GFAP, glial fibrillary acidic protein; CC, corpus callosum; CTX, cortex; HP, hippocampus; OPT, optic tracts.

GFAP-stained slides were scanned with a Zeiss Axio Scan.Z1 with Zen 2.5 blue edition software (Zeiss) and images of the corpus callosum (CC) and right and left optic tracts (OPT) and hippocampi (HP) were imported into ImageJ software. The measurement feature was employed to determine the mean gray density of areas of the CC, OPT and HP traced via freehand selection (Figure 1). An area with absence of immunostaining was selected from each section to remove background. Three sections per animal for each region were averaged to calculate average density values of GFAP staining (density = mean gray density – background). Assessments were performed by a single investigator blinded to the sex and injury condition of the images.

Cortical depth near the impact site was measured using methods described previously (15). The line tool in the Zen software was employed to draw a 1.75 mm horizontal line lateral from the superior sagittal fissure. A vertical line was then placed and measured at this location, tangential to the surface of the cortex and extending to the dorsal boundary of the corpus callosum (Figure 1, red dashed line, “d”). A 500 μm-wide region surrounding the vertical line, extending the depth of the cortex (CTX), was captured and imported into ImageJ for analysis of GFAP staining density as described.

### Statistical Analyses

Statistical analyses were performed with SPSS 21.0 (IBM Corp., Armonk, NY, USA) or SAS Studio 3.71 (SAS Institute Inc., Cary, NC, USA). Righting reflex and cortical depth data did not pass the homogeneity of variance test (Levene's test of the equality of variances) and were analyzed with Kruskal–Wallis tests and adjusted pairwise comparisons (SPSS). Apnea data were analyzed with a mixed model ANOVA (PROC MIXED; SAS) with sex as a fixed factor and day as a repeated measure. For body weight data and behavioral tests performed at multiple time points, mixed model ANOVAs (PROC MIXED) were performed with sex and injury as fixed factors and time as a repeated measures factor. Distance traveled data from open field testing were converted to natural log values to meet homogeneity of variance requirements. Tests performed at one time point (y-maze, EZM, MBT, TST) were analyzed with two-way ANOVAs (PROC GLM; SAS), with sex and injury as fixed factors. GFAP staining density in the CC was also analyzed with a two-way ANOVA (sex x injury); OPT, CTX, and HP staining density were analyzed with three-way ANOVAs, with sex and injury as fixed factors and side (left vs. right) as a repeated measure. CTX GFAP data were converted to inverse values to meet homogeneity of variance requirements. Where *post-hoc* testing was necessary, Bonferroni-corrected planned contrasts were performed. Following statistically significant main effects or planned contrasts, effect size (Cohen's *d*) was calculated as $\left|\frac{\mu_{1}-\mu_{2}}{s_{pooled}}\right|$, where s_pooled_ = $\sqrt{\frac{s_{1}^{2}+\;s_{2}^{2}}{2}}$. Figures were made using Microsoft Excel 2016 and Daniel's XL Toolbox 7.2.13, and data shown in all figures represent the means ± standard error of the means unless otherwise specified.

## Results

### Apnea and Righting Reflexes

**Figure 2:**
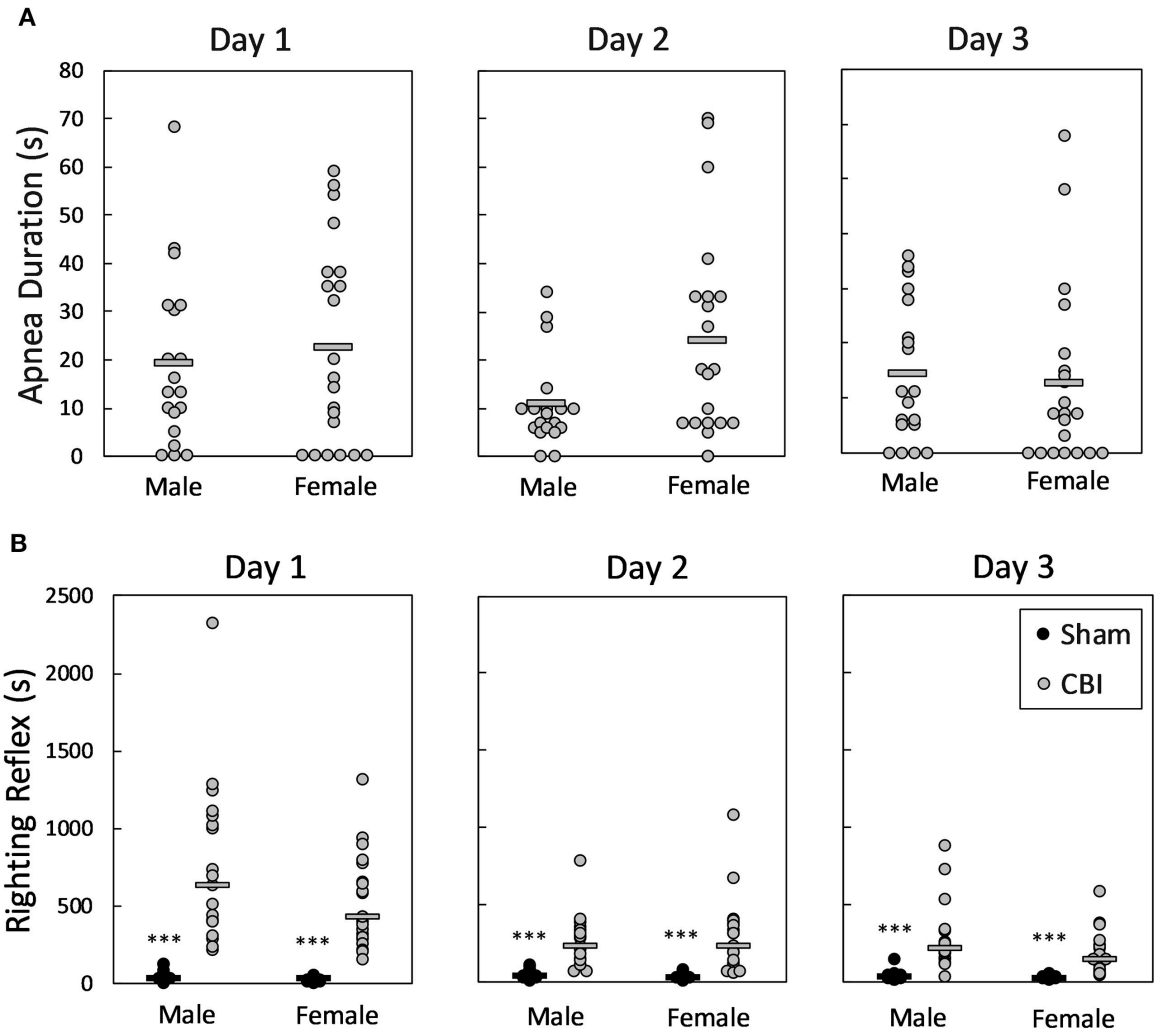
Apnea **(A)** and righting reflexes **(B)** following CBI on each injury day. Shown are individual data points; bars in **(A)** represent group means, in **(B)** represent group medians. The legend in lower right panel applies to all plots. There were no significant differences between male and female mice in the duration of apnea following CBI on any of the injury days **(A)**. On each of the injury days, mice sustaining injury had significantly longer righting reflexes than sham-treated mice of the same sex **(B)**. The asterisk (***) in **(B)** represents a significant effect of injury in the given sex, where CBI > Sham, *p* < 0.0001. There were no significant differences in righting reflex times between male and female mice, either injured or sham-treated. CBI, concussive brain injury.

Apnea was not observed in any mice following sham procedures; apnea in mice following CBI each day is shown in Figure 2A. Durations of apnea were equivalent between male and female mice and across injury days (*p* > 0.1406). Righting reflexes following sham and CBI procedures on the three injury days are shown in Figure 2B. Righting reflexes were longer in injured mice than in sham mice of the same sex on all three injury days [H(3) = 56.069, 50.479, 49.442, *p* < 0.0001 for injury days 1, 2, and 3, respectively]. There were no sex differences in righting reflexes in either the sham-treated mice or the injured mice on any injury days (adjusted *p* = 1.0).

### Body Weights

There was a significant sex by injury interaction effect on post-injury body weights [*F*_(1, 73.2)_ = 7.38, *p* = 0.0082]. Bonferroni-adjusted planned contrasts revealed that post-injury, the body weights of injured male mice remained lower than the weights of sham-treated male mice (*p* = 0.0064, *d* = 0.30), but injury did not affect body weights of female mice (*p* = 1.0; Table 1).

### Histological Findings

**Figure 3:**
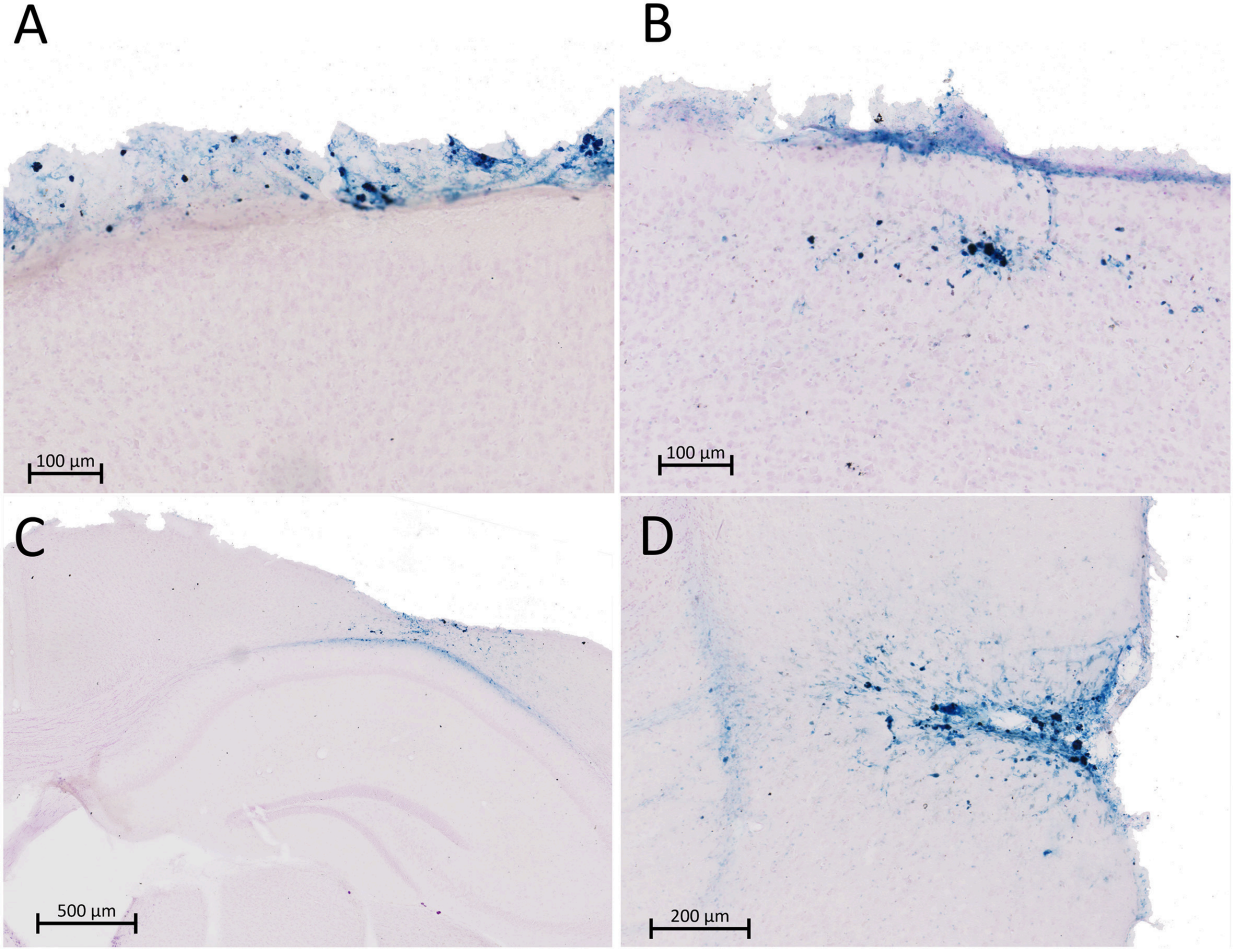
Prussian blue (PB) staining was performed in 29 mice for microbleeds after rCBI. Sections shown in **(A–D)** represent four different animals that stained positive for microbleeds. Eleven injured mice were negative for PB staining (not shown). Most mice positive for PB staining presented with microbleeds limited to the surface of the brain in the glia limitans (*n* = 11; **A**) or extending into the most superficial layers of the cortex (*n* = 5; **B**). A very limited number of animals had more severe injury involving the corpus callosum (*n* = 2; **C**) or temporal regions of cortex (*n* = 3; **D**). Two mice had both mild surface bleeds **(A)** and temporal bleeding **(C)**.

The brains from 29 injured mice and 10 sham mice underwent Prussian blue staining to detect microbleeds. Examples of Prussian blue staining in injured mice are shown in Figure 3. (No positive staining was seen in sham mice). Of the 29 injured mice, 11 of the mice were negative for Prussian blue staining (data not shown). Eleven animals (six females and five males) showed evidence of microbleeding only on the very surface of the brain near the impact site (Figure 3A), primarily observed in the glia limitans. An additional five mice (three males and two females) had surface bleeding with extension into cortical layers (Figure 3B), and two mice (one of each sex; Figure 3C) had more extensive injury, with evidence of cortex compression and bleeding that extended into the corpus callosum. Three more of the 29 mice, all female, had lateral/temporal bleeding near the rhinal sulcus as seen in Figure 3D. (Two mice had both mild surface bleeds and lateral bleeding). The more extensive injuries as assessed by Prussian blue staining were not associated with increased apnea or longer righting reflex times (data not shown).

**Figure 4:**
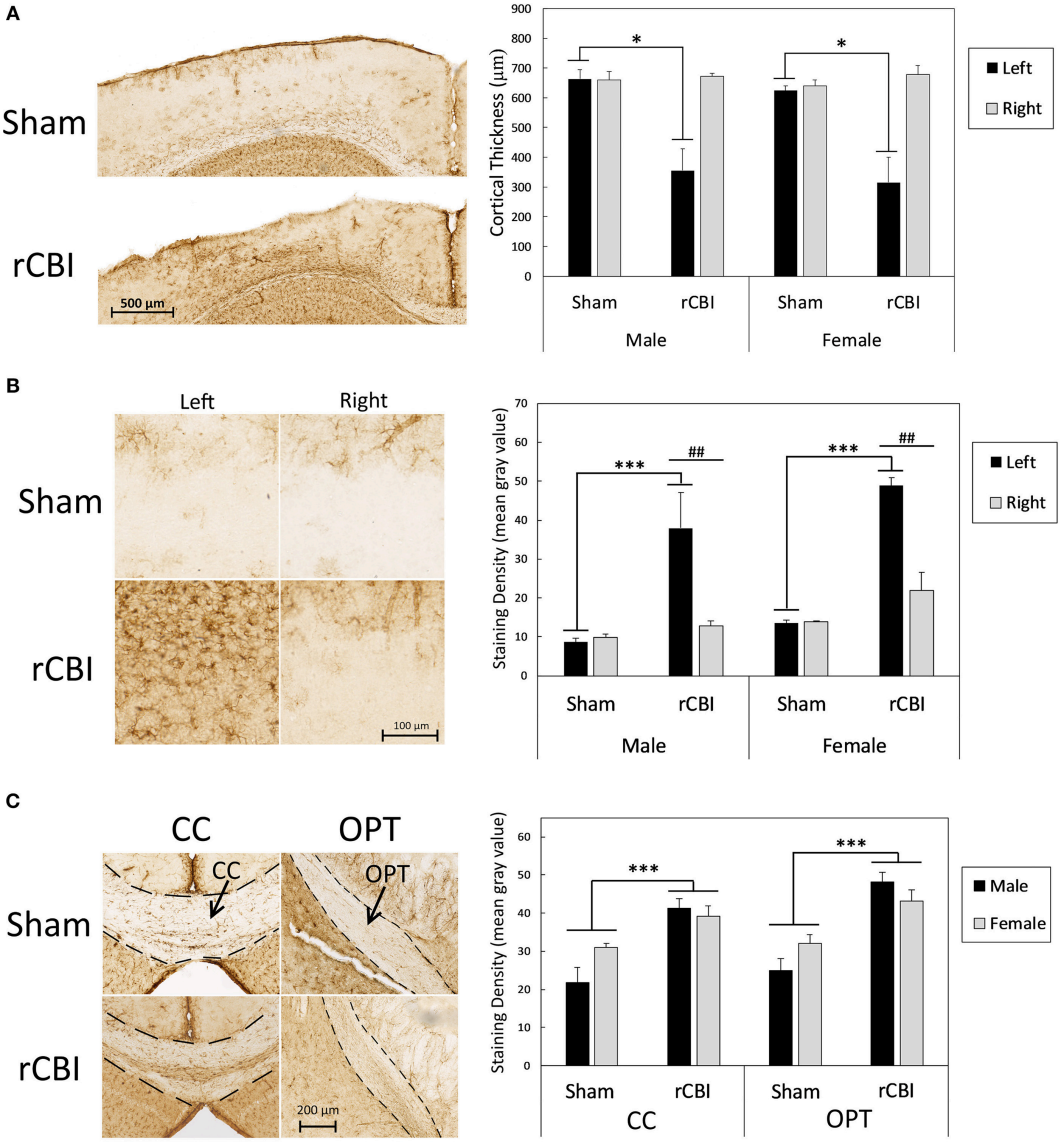
Cortical atrophy as measured by cortical thickness and astrogliosis following rCBI as evidenced by GFAP staining. Representative photomicrographs represent approximate median values. One year following injuries, injured mice had significantly thinner CTX at the injury site than sham-treated mice **(A)**. Asterisks (*) in **(A)** represent a significant main effect of injury in the represented sex (Sham > rCBI; *p* < 0.05). There was also significantly increased astrogliosis in the perilesional CTX on the injured (left) side of the brain compared to the right side and compared to the left side of sham mice **(B)**. Asterisks (***) in **(B)** represent a significant effect of injury on the left (injured) side of the brain (rCBI > Sham; *p* < 0.0001); pound signs (##) represent significant differences in staining density between the right (uninjured) and left (injured) sides of the brain in the represented sex (Left > Right; *p* < 0.01). GFAP staining density was significantly increased following injuries in white matter tracts, specifically the CC and OPT **(C)**. Asterisks (***) in **(C)** represent a significant main effect of injury (rCBI > sham) in the represented brain region (*p* < 0.001). rCBI, repetitive concussive brain injury; GFAP, glial fibrillary acidic protein; CTX, cortex; CC, corpus callosum; OPT, optic tracts.

Figure 4 is a summary of cortical atrophy, as measured by cortical thickness, and astrogliosis after rCBI. Analysis of cortical depth on the left side of the brain near the impact site showed a significant effect of group [H(3) = 16.786, *p* = 0.001]; both male and female injured mice had significantly thinner CTX at the injury site than respective sham controls (*p* = 0.021 and 0.026, respectively; Figure 4A). There was also increased GFAP staining density in the CTX beneath the impact site on the injured side of the brain [injury × side interaction effect: *F*_(1, 19)_ = 15.36, *p* = 0.0009; Figure 4B]. Astrogliosis was greater in the left CTX of injured mice than on the right side of injured mice (*p* = 0.0012, *d* = 1.71) and staining density in the injured left CTX was also greater than that in the left CTX of sham-control mice (*p* < 0.0001, *d* = 2.01). There was also a main effect of sex [*F*_(1, 19)_ = 12.10, *p* = 0.0025, *d* = 0.41], with female mice having greater levels of GFAP staining in the CTX than male mice (data not shown). Although there was an injury × sex interaction effect on GFAP staining in the HP [*F*_(1, 19)_ = 5.01, *p* = 0.0373], *post-hoc* tests did not reveal significant comparisons (male sham vs. male rCBI: *p* = 0.2248; female sham vs. female rCBI: *p* = 1.0; male rCBI vs. female rCBI: *p* = 1.0; male sham vs. female sham: *p* = 0.0896; data not shown).

However, rCBI increased astrogliosis in white matter tracts. Representative samples from each sex and injury group of GFAP staining in white matter tracts, the CC and OPT, are shown in Figure 4C. There was a significant main effect of injury on GFAP-immunostaining in the CC [*F*_(1, 20)_ = 25.23, *p* < 0.0001, *d* = 1.89]; mice that had sustained rCBI had greater GFAP-immunostaining in the CC than sham-treated mice. In the OPT, a three-way mixed-models ANOVA showed a significant main effect of injury on GFAP staining density [*F*_(1, 20.2)_ = 21.54, *p* = 0.0002, *d* = 1.79]. Injured mice had significantly greater amounts of GFAP staining in the OPT than sham-treated animals.

### Open Field

**Figure 5:**
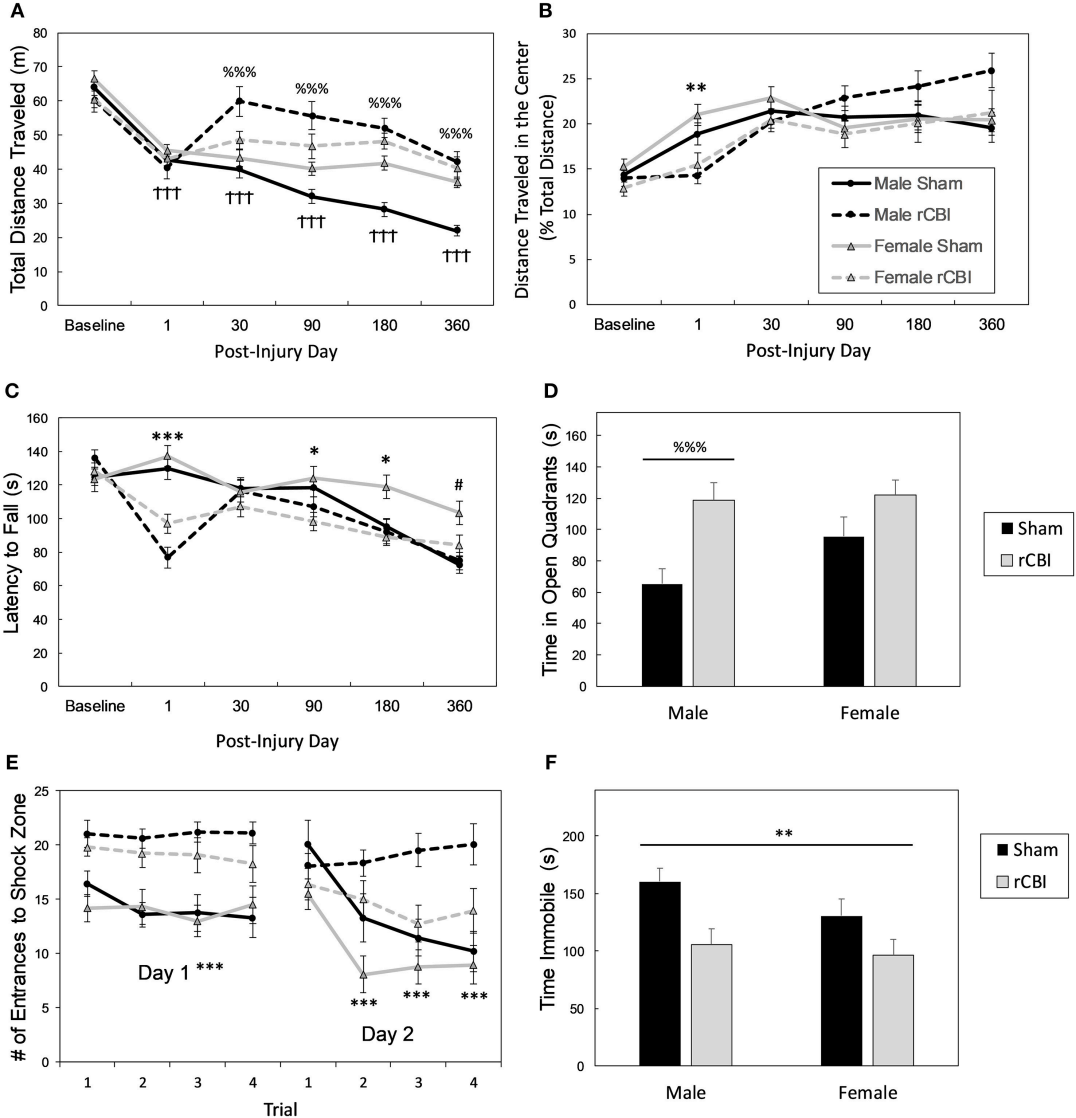
Chronic behavioral effects of rCBI. Legend in **(B)** applies to **(A,B,C,E)**. Behavioral testing following rCBI revealed functional deficits in the OF **(A,B)**, rotarod **(C)**, EZM **(D)**, APA **(E)**, and TST **(F)** tests. Injured male mice began exhibiting a hyperactive phenotype in the OF about a month following injuries **(A)**. All injured mice were significantly less active in the center zone of the OF the day following the final injury (expressed as a percentage of total activity), suggesting anxiety **(B)**. On the rotarod **(C)**, there were significant motor deficits the day following the injuries; slight impairment remained for up to 6 months. Only injured male mice had significantly different behavioral performance in the EZM (1 year; **D**); they spent increased time in the bright and exposed quadrants compared to sham-treated mice. One year post-injuries, all mice had significant cognitive deficits as assessed on the APA test **(E)**, and showed significantly greater agitation (less immobility) in the TST for depressive behaviors **(F)**. The cross signs († † †; *p* < 0.0001) in **(A)** indicate a significant effect of testing day in female mice; female mice became less active in the OF arena following baseline testing. The percent signs (%%% *p* < 0.0001) in **(A,D)** represent an injury effect in male mice only where Injured > Sham on the measured behavior. Asterisks (*p < 0.05, **p < 0.01 or ***p < 0.0001 in **B,C,E,F**) represent a main effect of injury on the given behavior (C: rCBI < Sham; E: rCBI > Sham); The pound sign (# *p* < 0.05) in **(B,C,F)** represents a main effect of sex (female > male). rCBI, repetitive concussive brain injury; OF, open field; EZM, elevated zero maze, APA, active place avoidance; TST, tail suspension test.

In the OF, there was a significant injury × sex × day interaction effect for the distance traveled in the arena [*F*_(5, 354)_ = 7.55, *p* < 0.0001] (Figure 5A). Separate two-way ANOVAs (injury × day) were performed for each sex. In males, there was a significant injury × day interaction effect [*F*_(5, 179)_ = 22.98, *p* < 0.0001]; sham-treated and injured male mice were equally active in the OF during baseline testing and on the day following the final injury (*p* = 1.0), but injured male mice ambulated greater distances on all subsequent testing days compared to sham-treated male mice (days 30, 90, 180, and 360; *p* < 0.0001; *d* = 1.28, 1.47, 2.06, 2.02, respectively). In females, there was only a main effect of day [*F*_(5, 175)_ = 28.29, *p* < 0.0001]; the female mice were less active in the arena on all subsequent days compared to baseline testing (*p* < 0.0001, *d* = 1.71, 1.52, 1.58, 1.56, 2.46 for days 1, 30, 90, 180, and 360, respectively), and at the 1-year time point the female mice were less active when compared to day 1 (*p* = 0.0078, *d* = 0.62), day 30 (*p* = 0.0001, *d* = 0.87), and day 90 (*p* = 0.0054, *d* = 0.71).

There was an injury × day interaction effect on the distance traveled in the center zone of the OF (expressed as a percentage of the total distance traveled) [*F*_(5, 355)_ = 7.03, *p* < 0.0001; Figure 5B]. On the day following the final injury, injured mice were less active in the center of the arena than sham controls (*p* = 0.0018, *d* = 0.98). The effect on day 360 neared significance (*p* = 0.0990, *d* = 0.44), where injured mice were more active in the center of the arena.

### Rotarod

For rotarod testing (Figure 5C), there was a significant injury by day interaction effect [*F*_(5, 355)_ = 11.96, *p* < 0.0001] and a significant sex by day interaction effect [*F*_(5, 355)_ = 3.93, *p* = 0.0017]. Planned Bonferroni-corrected comparisons showed that there was a main effect of rCBI on rotarod performance on days 1 (*p* < 0.0001, *d* = 1.65), 90 (*p* = 0.0138, *d* = 0.73), and 180 (*p* = 0.0402, *d* = 0.57) following injury, with mice sustaining rCBI falling from the rotarod at shorter latencies than sham controls. Also, planned contrasts revealed that overall, female mice stayed on the rotarod longer than male mice at the 1-year time point (day 360; *p* = 0.0096, *d* = 0.78).

### Elevated Zero Maze, Marble Burying Test, y-Maze

There was an injury by sex interaction effect on anxiety-like behaviors tested in the EZM 1 year following rCBI [*F*_(1, 68)_ = 6.31, *p* = 0.0144; Figure 5D]. Bonferroni-corrected planned contrasts showed that sham-treated females and injured females spent equivalent amounts of time in the open quadrants of the maze (*p* = 1.0), but injured male mice spent significantly more time in the open quadrants than the male sham-treated mice (*p* < 0.0001, *d* = 1.48). There were no effects of injury or sex on the number of marbles buried in the MBT (*p* > 0.2966; data not shown). Likewise, there were no effects of injury or sex, or interaction between the two factors on the percent of time spent in the novel arm in the y-maze test [*F*_(1, 67)_ < 0.942, *p* > 0.335; data not shown].

### Active Place Avoidance

There was a main effect of sex on distance traveled during the 10-min acclimation trial of the APA test [*F*_(1, 66)_ = 9.11, *p* = 0.0036, *d* = 0.73], with females ambulating greater distances in the arena (data not shown). A mixed-models analysis (injury × sex × trial) showed a significant main effect of injury on spatial learning in the APA test on the first day of testing [*F*_(1, 49.4)_ = 31.23, *p* < 0.0001, *d* = 1.34; Figure 5E], and an injury x trial interaction effect on the second day when the shock zone was relocated [*F*_(3, 133)_ = 9.89, *p* < 0.0001]. Sham-treated mice had fewer entrances to the shock zone of the apparatus on all trials on the first day of testing demonstrating quicker learning of the location of the zone; on the second day, injured mice had equal performance to sham controls on the first trial, but shams had fewer entries to the shock zone on the remaining three trials (*p* < 0.0001; *d* = 1.33, 1.53, 1.62 for trials 2, 3, and 4, respectively).

### Tail Suspension Test

There was a significant effect of injury on immobility in the TST for depressive-like behaviors [*F*_(1, 67)_ = 10.62, *p* = 0.0018, *d* = 0.78; Figure 5F]; sham-control mice spent greater time in an immobile state than injured mice.

## Discussion

### Summary of Pathological and Behavioral Findings Following rCBI

This study employed a mouse model to study the effects of repetitive concussive brain injuries on behavior and brain pathology in both male and female animals up to 1 year following injuries. A summary of significant findings is found in Table 2. Cortical atrophy was found at the lesion site (Figure 4A); our previous study in which mice were euthanized at a more acute time point (32 days) found equal cortical thickness (15), suggesting this is a chronic, degenerative process rather than an immediate effect of the impacts. GFAP staining was unaffected by injury in the hippocampus but increased in the perilesional cortex and axonal tracts (corpus callosum and optic tracts) in both male and female mice (Figure 4B). In addition, Prussian blue staining (Figure 3) showed that the injuries led to cerebral microbleeds primarily limited to the site of the injury and the cortex beneath, consistent with clinical findings following mild TBI [e.g., (37, 38)] and with prior rCBI studies in mice (39, 40). A subset of the mice also had positive Prussian blue staining distal from the primary injury site near the rhinal sulcus; Sauerbeck and colleagues recently reported pathology in similar locations following concussive-rotational injury (CHIMERA) (41).

**Table 2:** Summary of significant findings between injured and sham mice, or main effects of sex.

**Measure**	**Significant effect**	***p*-value**	**Effect size (Cohen's *d*)**
Post-injury weights (% baseline)	Male Sham > Male rCBI	*p* = 0.0064	*d* = 0.30
Righting reflex, Injury Days 1-3	rCBI > Sham	*p* < 0.0001	N/A
Cortical thickness	rCBI < Sham	*p* < 0.05	N/A
GFAP staining density			
Cortex (male vs. female)	Female > Male	*p* = 0.0025	*d* = 0.41
Cortex (injured (left) side of brain)	rCBI > Sham	*p* < 0.0001	*d* = 2.01
Corpus callosum	rCBI > Sham	*p* < 0.0001	*d* = 1.89
Optic tracts	rCBI > Sham	*p* = 0.0002	*d* = 1.79
Rotarod, Latency to Fall			
Day 1	Sham > rCBI	*p* < 0.0001	*d* = 1.65
Day 90	Sham > rCBI	*p* = 0.0138	*d* = 0.73
Day 180	Sham > rCBI	*p* = 0.0402	*d* = 0.57
Day 360	Female > Male	*p* = 0.0096	*d* = 0.78
OF, Distance traveled; Males			
Day 30	Male rCBI > Male Sham	*p* < 0.0001	*d* = 1.28
Day 90		*p* < 0.0001	*d* = 1.47
Day 180		*p* < 0.0001	*d* = 2.06
Day 360		*p* < 0.0001	*d* = 2.02
OF, Center distance traveled (% Total); Day 1	Sham > rCBI	*p* < 0.0018	*d* = 0.98
EZM, Time in open quadrants (1 year)	Male rCBI > Male Sham	*p* < 0.0001	*d* = 1.48
APA, Acclimation, Distance traveled (1 year)	Female > Male	*p* = 0.0036	*d* = 0.73
APA, # of entries to shock zone; Day 1 (1 year)	rCBI > Sham	*p* < 0.0001	*d* = 1.34
APA, # of entries to shock zone; Day 2 (1 year)			
Trial 2	rCBI > Sham	*p* < 0.0001	*d* = 1.33
Trial 3		*p* < 0.0001	*d* = 1.53
Trial 4		*p* < 0.0001	*d* = 1.62
TST, Time immobile (1 year)	Sham > rCBI	*p* = 0.0018	*d* = 0.78

*Shown are the dependent measures in the current experiment for which significant effects of injury or sex were found. P-values from ANOVAs (or Bonferroni-corrected post hoc comparisons) or Kruskal–Wallis tests, and calculated Effect sizes (Cohen's d) are provided. Cohen's d = $\left|\frac{\mu_{1}-\mu_{2}}{s_{pooled}}\;\right|$*.

Behaviorally, both male and female mice had motor impairments on the rotarod for up to 6 months following the concussions (Figure 5C), and both sexes had significant cognitive deficits at the 1-year time point on the APA task (Figure 5E). Injured male mice showed some behavioral differences compared to sham controls that were not seen in female mice: in the OF the injured male mice were hyperactive on post-injury days 30 and beyond (Figure 5A), and in the EZM 1 year following injury they spent greater amounts of time in the open/exposed quadrants (Figure 3D). Additionally, male mice sustained weight loss as a result of the injuries (Table 1), and their weights remained lower compared to uninjured mice for the duration of the study.

### Axonal Injury and CBI

Axonal injury is a prominent feature of CBI, both clinically and in animal models (42, 43), and the CC may be particularly vulnerable to injury. Activated microglia, together with axonal degeneration and atrophy in the CC have been described in chronic (up to 18 years post-injury), but not acute, TBI patients (44). In a mouse model of single CBI, Marion and colleagues recently described axonal damage in the CC, including degenerating and demyelinated axons, disruption of paranodes where myelin attaches to axons, and overall CC atrophy at 8 weeks post-injury (45). Wild-type mouse models of rCBI describing axonal damage and/or neuroinflammation in the CC and other axonal tracts at more acute time periods are numerous (11–15, 40, 46–50). Gold and colleagues recently reported a decrease in CC volume 6 months following 10 CBIs (16), and there are a smaller number of studies that have extended observations to more chronic time points: CC atrophy as measured by thickness has been reported in mouse rCBI models 1 (17) and 2 (19) years following the injuries. These changes were paralleled by increases in the CC in markers for astrocytes (GFAP) and microglia (Iba1) (16, 17, 19), indicative of neuroinflammation, consistent with the current data.

### Changes in Spontaneous Activity and Motor Behaviors Following rCBI

During the year of study, the mice had significant motor deficits on the rotarod the day following the final injury but also remained slightly impaired up to 6 months following injuries, and male mice developed a hyperactive phenotype in the OF after 1 month. Rotarod deficits have been reported up to 1 year following 30 closed head injuries in mice (18). Motor deficits following experimental TBI are often acute and relatively transient compared to cognitive deficits [e.g., (14, 16, 51)] and here we show that motor deficits are resolved while cognitive deficits are observed 1 year following injuries. Hyperactivity in male mice has been previously reported in rCBI models (52, 53), as well as in more severe injury models such as controlled cortical impact (CCI) that result in overt damage to the hippocampus (32, 54–59). Ultimately, there are a variety of experimental manipulations that can result in increased locomotion, and it has been suggested that any lesion or damage involving a complex control system that modulates and suppresses activity, located along the axis between the entorhinal cortex and olfactory bulb, may result in hyperactive behavior (60).

### Effects of rCBI on Cognition

Consistent with prior long-term rCBI studies that employed the Barnes maze as a spatial cognitive test 1 year or longer post-injuries (17, 19), all injured mice in this study had significant cognitive deficits as assessed in the APA test (a test of spatial learning and memory), despite showing no deficits on the y-maze test of novel arm recognition (a test of spatial episodic memory). Deficits in spatial learning and memory are most often attributed to hippocampal damage and dysfunction, but in the current study parietal association cortical atrophy and astrogliosis may have been a factor as the parietal cortex is involved in linking motion and visual information during the early steps of map formation in spatial tasks (61). The APA test has been shown to detect cognitive deficits following single and rCBI in mice at a more acute time period (12) and for up to 12 months following repetitive mild TBI in a human tau-expressing transgenic mouse model (62); it is a particularly difficult task as it requires the animal to attend only to stationary visual room cues, ignoring the olfactory cues on the rotating apparatus and to continue to move with the arena in order to avoid the shock zone (63). In addition, two intact hippocampi and the fimbria containing the hippocampal commissural fibers (a white matter axonal tract) are required for successful performance of the task (64). Thus, our disparate findings on the y-maze and APA are likely due to test difficulty but could also relate to test modality. Sangobowale and colleagues recently demonstrated significant deficits on the APA task 8 days following a single CBI in mice, an injury that was characterized by reduced myelin in the CC (and other white matter regions) as assessed by Luxol fast blue. Both the cognitive deficits and axonal damage were reduced by treatment administered 12 h following injury with a combination of minocycline, an agent that inhibits microglial activation and prevents myelin loss, and N-acetylcysteine, an antioxidant drug with anti-inflammatory actions (35). These results suggest that cognitive deficits associated with damage in white matter tracts may serve as useful functional and neuropathological endpoints for assessing delayed effects of potential therapeutic agents.

### Chronic “Emotional Dysregulation” in Rodent Models of rCBI

Finally, we employed the EZM and TST, respectively, to assess chronic neuropsychiatric symptoms following rCBI, as anxiety and depression are among the most common long-term complaints in clinical TBI populations. Injured male, but not female, mice spent increased amounts of time in the bright and open regions of the maze compared to sham controls, suggesting reduced anxiety, consistent with other studies 1 year following rCBI in mice (17, 19), but see (18), or at more acute time periods (16, 53, 65). A curious finding of this study is increased amount of agitation (less immobility) in the TST of the injured mice compared to the sham controls. Greater amounts of immobility in this test [and in a similar test, the forced swim test (FST)], are interpreted as a state of despair or “depression” in animal models (66, 67). Although they found no differences in the TST, Gold and colleagues reported greater amounts of “highly mobile” time in the FST in a murine rCBI model with CC atrophy, microgliosis, and astrogliosis 6 months following injury (16). This finding was interpreted as a symptom of “emotional dysregulation,” and in our model, together with hyperactivity in the OF and the increased amount of time in the open quadrants of the EZM, could suggest a pathological phenotype of agitation and risk-taking. However, these findings do not model the more common clinical symptoms of depression and anxiety that are diagnosed following TBI. Overall it has been noted that compared to motor and cognitive deficits that are employed in pre-clinical studies, findings regarding neuropsychiatric symptoms have been much more inconsistent and that further study, as well as the inclusion of different functional testing models, is needed (33, 67, 68).

### Sex Differences in Functional Deficits Following TBI

Injured female mice in this study fared better than males. Unlike injured male mice their weights remained at the same level as sham controls, they did not exhibit the hyperactive phenotype in the OF, and they had normal behavior in the EZM. Chronic injury-induced astrogliosis cannot explain the long-term sex-differences in behavior in this study, as males and females showed the same neuropathological profile at the 1-year time point. However, we previously showed that male mice have increased astrogliosis compared to females at a more acute time point (~1 month) following rCBI (15). Translational TBI studies are becoming more inclusive of both sexes, and there is mounting evidence that female rodents may fare better acutely (within days following injury) in measures of behavior and neuroinflammation than males (15, 30, 69–75). As noted in a recent review (76), there are also many reports showing no sex differences or that males have better outcomes than females [e.g., (32, 76–78)], and definitive conclusions from rodent studies are difficult at this point as investigators are using different species at different ages, different functional testing paradigms at varying times following TBI, and different injury models. However, although phase III clinical trials of progesterone to date have failed, the neuroprotective effects of estrogen and progesterone are well-established in animal models of neurotrauma (79–81), and it is possible that these hormones are exerting long-term protective/therapeutic effects in this model via biological mechanisms that were unexplored in this study. In addition to developmentally programmed sex differences, the on-going effects of sex hormones in the brain are far-reaching. Sex hormone receptors are located in glial cells and throughout neurons, where they have many actions, including regulation of signaling pathways and direct and indirect effects on gene expression, leading to alteration of many physiological and behavioral functions (82). Although the initial focus of studies on sex hormones and behavior focused on the hypothalamus and sexual behaviors, it is now realized that steroids have more global effects on the brain, including regions such as the hippocampus, prefrontal cortex, cerebellum, and periacquadectal gray, resulting in sex differences in addiction, responses to stress, mood regulation, and pain sensitivity, among others (82). Ultimately, the potential effects of steroid hormones following injury, both neural and glial, genomic and non-genomic, are complex and will require dedicated, targeted study.

## Conclusions

In summary, this study employed a moderately severe chronic rCBI model in wild-type mice. Consistent with reports of other investigators (17–19), there were significant behavioral deficits concurrent with ongoing neuroinflammation in axonal tracts 1 year following the injuries, Furthermore, this is the first long-term rCBI study in rodents that has been inclusive of both sexes, and we have demonstrated that male mice fare worse on two behavioral tasks, despite showing a similar neuropathological profile to injured female mice. There is a need for continued development of translational models of chronic rTBI with measurable functional and pathological features that lend themselves to therapeutic intervention.

## Ethics Statement

All animal procedures were approved by the Institutional Animal Care and Use Committee at the Uniformed Services University of the Health Sciences (Bethesda, MD) and the mice were housed in Association for Assessment and Accreditation of Laboratory Animal Care-Accredited facilities.

## Author Contributions

LT and JM designed and planned the study. TBI procedures were performed by AF. Behavioral testing was performed by LT and AV. Immunohistochemical processing and analyses were performed by LT, AF, and AV. Data analyses were performed by LT. Final editing of the manuscript by LT and JM.

### Conflict of Interest Statement

The authors declare that the research was conducted in the absence of any commercial or financial relationships that could be construed as a potential conflict of interest.
